# Factors associated with health state valuations: a secondary analysis of an EQ-5D-3L valuation study from Jordan

**DOI:** 10.1007/s11136-025-04151-2

**Published:** 2026-05-03

**Authors:** Abeer Al Rabayah, Fatima Al Sayah, Marjan Arvandi, Uwe Siebert

**Affiliations:** 1 Institute of Public Health, Medical Decision Making and Health Technology Assessment, Department of Public Health, Health Services Research, and Health Technology Assessment, UMIT TIROL - University for Health Sciences and Technology, Hall in Tirol, Austria; 2 Center for Drug Policy and Technology Assessment, Pharmacy Department, King Hussein Cancer Center, Amman, Jordan; 3 Alberta PROMs & EQ-5D Research & Support Unit (APERSU), School of Public Health, University of Alberta, Edmonton, AB Canada; 4 Division of Health Technology Assessment, ONCOTYROL - Center for Personalized Cancer Medicine, Innsbruck, Austria; 5 Center for Health Decision Science, Departments of Epidemiology and Health Policy & Management, Harvard T.H. Chan School of Public Health, Boston, MA USA; 6 Harvard Medical School, Institute for Technology Assessment and Department of Radiology, Massachusetts General Hospital, Boston, MA USA

**Keywords:** Valuation, cTTO, Jordan, Predictors, EQ-5D-3L

## Abstract

**Purpose:**

Elicitation of EQ-5D value sets using time trade-off (TTO) is based on the general population’s degree of preference for specific health states. This study aimed to identify factors associated with health state valuations among the Jordanian population, conducted as part of the EQ-5D-3L valuation study.

**Methods:**

A cross-sectional study using EQ-5D-3L valuation data from Jordanian participants was used to identify factors associated with health state valuation. The valuation study followed the latest EuroQol Group valuation protocol (EQ-VT v2.1). Participants aged ≥ 18 years were interviewed via videoconferencing. Sociodemographic, geographic regions, and health indicators data were collected. We applied a quota sampling based on region, gender, and age. Univariate and multivariable (multilevel mixed effects model, Tobit random, and random intercept) regression models were applied to identify associations. Statistical analysis was conducted using STATA version 17.

**Results:**

From September 2021 to April 2022, 301 participants were included in the study, covering all Jordanian governorates from the north, middle, and south. A representative sample was achieved. 51% of the participants were females with a mean age of 41 (± 15 (SD)) years. Overall, 69% were married, 50% of the respondents had a higher education degree, and 77% were insured. The mean EQ VAS score was 81(± 14). The multilevel mixed effects model was the best-fit model. Unemployment status and having more than one comorbidity were identified as statistically significant factors associated with cTTO health state valuation in Jordan.

**Conclusion:**

Socioeconomic characteristics have limited ability to explain how Jordanians value cTTO health states. Future research should explore cultural dimensions and personality traits to understand health state valuations better.

**Electronic supplementary material:**

The online version of this article (10.1007/s11136-025-04151-2) contains supplementary material, which is available to authorized users.

## Introduction

Health outcomes are essential for evaluating the effectiveness of medical interventions [1]. Measuring clinical health outcomes—such as disease progression or mortality—using defined endpoints and specific measurement methods is crucial in clinical decision-making [1–3]. However, measuring such clinical health outcomes alone might not be sufficient for clinical guideline development and resource allocation, assigning value to these outcomes based on patients’ preferences is necessary for informed decision-making.

Despite the complexity of valuing health outcomes, valuation methods offer a practical approach to assigning values to health states, aiding decision-makers and clinicians in valuing patient-reported outcomes in clinical trials and routine practice. In addition, health outcomes valuation addresses the challenge of translating health benefits into monetary terms. It provides a methodological framework for conducting cost-utility analysis (CUA), a type of cost-effectiveness analysis that evaluates costs monetarily and outcomes in quality-adjusted life years (QALYs) [2–5]. Using QALYs incorporates the valuation of measured health outcomes using utility scores to adjust survival[2, 8].By integrating morbidity, mortality, and health states values, QALYs offer a comprehensive measure of health outcomes [2–8].

Health state valuation is fundamentally dependent on people’s preferences. Patients or the general population may do the valuation task. Consequently, tools used to measure health state values must be preference-based. Preference-based quality of life indices – so called utilities—can be elicited directly or indirectly. Direct methods include the Time Trade-Off (TTO), Standard Gamble, and Visual Analog Scale[3, 5] while indirect methods use Generic Preference-Based Measures (GPBMs)[3, 5]such as the EQ-5D [9, 10], the Short Form 6 dimension (SF-6D) [11], Quality of Life (AQoL)-8D [12], and the Health Utilities Index version 3 (HUI3) [13]. Among these, EQ-5D is the most widely used GPBMs [14], with valuation following the EuroQol Group protocol (EQ-VT v2.1) [15, 16]. Numerous EQ-5D valuation studies have been conducted globally, with an increasing number in the Middle East and North Africa (MENA) region over the past four years, all using the general population for value elicitation.

Since health state valuation depends on people’s preferences, it is essential to explore whether demographics and socioeconomic status are associated with these valuations. Studies from outside the MENA region present mixed findings on these associations, with some identifying demographic and socioeconomic predictors of health state values, while others find no significant impact [17–20]. In the MENA region, only one study, conducted in Egypt, has identified determinants of health state valuation, specifically noting age, gender, and marital status [18]. However, it remains unclear whether similar determinants or factors apply to the Jordanian context. Furthermore, the Jordanian EQ-5D-3L valuation study was conducted during the COVID-19 pandemic[25], enabling us to assess whether COVID-19 infection is associated with health state valuation. Additionally, previous studies did not examine the impact of lifestyle factors, such as smoking and physical exercise, on the valuation of health states.

Generating empirical evidence on which factors are associated with health state valuations can guide sample selection in future health state valuation studies in the MENA region. Additionally, given the cultural and social values context in Jordan [21–24], this study may uncover unique factors specific to the MENA region. Therefore, our study aimed to identify factors associated with EQ-5D-3L health state valuation among the Jordanian population using the composite time trade-off (cTTO) method. This study is a secondary analysis of the EQ-5D-3L valuation study, and was not initially designed to test predictors of health state valuation nor to develop and validate a prediction model as used, for example, in prognosis.

## Methods

### Study design

We conducted a cross-sectional study using data from the EQ-5D-3L valuation survey [25] to identify sociodemographic, geographic, and health-related factors associated with health state valuation in Jordan. The valuation study followed the EQ-VT v2.1 protocol established by the EuroQol Group [15, 16]. The EQ-5D-3L is a generic preference-based measure of health state utilities[26]. It consists of a descriptive system that includes five health dimensions: mobility, self-care, usual activities, pain/discomfort, and depression/anxiety[26]. Each dimension has three severity levels (1: no health problems, 2: moderate health problems, 3: extreme health problems). The selection of severity level for each health problem generates a five-digit number (e.g., 12,233) that is translated into a utility index score using the accompanying EQ-5D-3L value set [26].

Three hundred and one participants aged 18 years and above were interviewed via videoconferencing. Sociodemographic and other health indicators data were collected during the valuation study[25]. The study implemented quota sampling according to region, gender, and age, using the national population census from the Jordanian Department of Statistics (DOS) to achieve national representativeness [27, 25]. A global marketing research company was tasked to recruit participants[25].

### Ethical approval

The study was reviewed and approved by the Institutional Review Board (IRB) at the King Hussein Cancer Center, Amman, Jordan (Approval number: 21 KHCC 054, 18 May). Additional approval was granted by the Research Committee for Scientific Ethical Questions (RCSEQ) at UMIT TIROL—University for Health Sciences and Technology, Hall in Tirol, Austria (Approval number: 2986, 19 October 2021).

### Data collection

Data for the valuation survey were collected between 28 September 2021 and 2 April 2022. During this period, respondents also completed the EQ-5D-3L health profile description and the EQ VAS [25, 26].

Participants also provided sociodemographic and health indicators information, which served as independent variables (factors) in this study (Table 1). Given Jordan’s high rates of obesity and smoking, additional questions were included to assess the potential impact of smoking and exercising on health state valuation [29, 30]. Moreover, as the study was conducted during the COVID-19 pandemic, participants were asked about their COVID-19 infection and vaccination status to examine potential associations between the pandemic experience and health state valuation.

**Table 1:** Characteristics of study participants [25]

Characteristic	Overall (N = 301) [Mean (± SD) or n (%)]
**Age, years**	41 (± 15)
18–30	105(35)
31–45	84(28)
46–60	75(25)
> 60	37(12)
**Gender, Female**	147 (49)
**Geographical region**	
North region (Irbid, Al-Mafraq, Jerash, Ajloun)	89 (30)
Middle region (Amman, Zarqa, Balqa, Madaba)	189(63)
South region (Al-Kerak, Aqaba, Ma’an, Tafilah)	23 (8.0)
**Region type-urban**	279 (93)
**Marital status**	
Single	78 (26)
Married	207 (69)
Divorced/widowed	16 (5.0)
**Education Status**	
Less than secondary	21 (7.0)
Secondary	79 (26)
Intermediate diploma	51 (17)
Bachelor’s degree and above	150 (50)
**Employment**	
Employed	154 (51)
Public sector	53 (34)
Private sector	80 (52)
Non-profit organizations	10 (7.0)
Self-employed	11(7.0)
Unemployed	105 (35)
Retired	42 (14)
**Health insurance**	
Have insurance	231(77)
Ministry of Health	99 (43)
Royal Medical Services	53 (23)
Universities’ Hospitals	10 (4.0)
Private insurance	46 (20)
Others	23 (10)
No insurance	70 (23)
**Number of comorbidities**	
None	218 (72)
1	49 (16)
2 or more	34 (11)
**Exercise status**	
Yes	171(57)
No	130 (43)
**Reported smoking status**	
Yes	121(40)
No	180 (60)
**Reported Covid-19 infection status**	
Yes	121(40)
No	180 (60)
**Reported Covid-19 vaccination status**	
Yes	277 (92)
No	24 (8.0)

SD: standard deviation, comorbidities: noncommunicable diseases like cardiovascular diseases, diabetes, asthma, and cancer, exercise status performing physical activity for a duration (less than 1 hour, 1–3 hours, or more than 3 hours per week.

The dependent variable in this study was the (cTTO) score, a direct utility measure anchored at zero (dead) with one representing perfect health[15, 16]. For health states considered worse than death, utility scores can take negative values [15, 16]. Each respondent provided valuations for ten health states, with cTTO utility scores generated according to the EuroQoL protocol [15, 16]. These scores, directly elicited through cTTO tasks, served as the dependent variable in our analysis. Detailed information about the cTTO task is available in prior publications [15, 16].

### Statistical analysis

Our statistical analysis framework comprised several types of analysis, including descriptive analysis, checks for model assumptions (multicollinearity among factors, normality of the residual distribution, and homoscedasticity), purposeful selection of factors for inclusion in multivariable models, testing of several regression models, and selection of the final model.

We conducted descriptive analyses to compute means, medians, standard deviations (SDs), interquartile ranges (IQRs), and frequencies for respondents’ sociodemographic, geographic, and health-related factors. Self-reported EQ VAS scores and health profiles were also analyzed descriptively.

We tested multicollinearity among potential factors using a correlation matrix, variance inflation factor (VIF), and tolerance values. Factors with VIF < 10 and tolerance > 0.1 were considered to have low multicollinearity. We also visually and statistically assessed the normality of the residual distribution using the Shapiro–Wilk test. The assumption of normality holds if the p-value is > 0.05. Also, we tested the homoscedasticity assumption using the Breusch-Pagan test. If the p-value is > 0.05, then the variance is constant, and homoscedasticity holds.

Given the continuous nature of cTTO responses, the nested structure of the data (each respondent completed 10 tasks) [31], and the heterogeneity of participants’ preferences, we tested three regression models to identify the best fit: random intercept model, multilevel mixed effects model, and Tobit random intercept model (to account for potential censoring at − 1, as respondents could not trade off more than 20 life years to avoid a health problem state) [32].

### Purposeful selection

We applied a purposeful selection method to select factors that should be included in the initial multivariable model. For factor selection, we used both p values (for statistical significance) and the change in estimate criterion (to appropriately adjust for confounding) [33] in two sequential steps.

First, we performed a univariate analysis to assess the impact of each factor on cTTO utility, adjusting for health state severity [17, 34] using the Level Sum Score (LSS) [32]. The LSS, ranging from 6 to 15, was calculated by summing levels across the EQ-5D-3L health dimensions [32]. We applied univariate analysis using the three models (multilevel mixed effects, random-intercept, and Tobit random-intercept) with factors showing p-values ≤ 0.25, selected for inclusion in the initial multivariable regression models [32, 35–38].

Second, we fit multivariable models corresponding to each model type (multilevel mixed effects, random-intercept, and Tobit random-intercept), incorporating all identified factors associated with cTTO from the univariate analysis. We removed statistically non-significant variables (*p* > 0.10) one at a time. However, we retained variables if they were potential confounders based on the change in estimate criterion. For this purpose, we defined a variable being a confounder if its removal from the multivariable model changes the coefficient of any other included variables by ≥ 15%.

The final multivariable regression models assessed the impact of factors, identified through purposeful selection, on the cTTO utility score, adjusting for health state severity using LSS. Statistical significance was set at *p* < 0.05. All analyses were conducted using STATA/BE version 17 [39].

### Model selection

The goodness of fit of the three final multivariable models was compared using the Akaike Information Criterion (AIC) and the Bayesian Information Criterion (BIC). Model selection was based on goodness of fit criteria (the model with the lowest AIC/BIC) [40].

## Results

### Respondents’ characteristics

A total of 301 participants, representing all geographic regions of Jordan (north, middle, and south), were included in the study. The sample was representative by region, age, and gender (Table 1). Participants had a mean age of 41 years (SD ± 15), with 51% identifying as female. The majority were married (69%), 50% held a higher education degree (bachelor’s and above), and 51% were employed (Table 2) [25].

**Table 2:** Valuation study national representativeness [25]

Characteristic	Sample Number and percent(n,%)	*Jordanian general population (%)	Percentage difference (%)
**North geographical region **			
Irbid	61(20.27)	20.00	+0.27
Al-Mafraq	13(4.32)	5.00	0.68
Jersah	9(2.99)	3.00	0.01
Ajloun	6(1.99)	2.00	0.01
**Middle geographical region**			
Amman	121(40.20)	39.00	+1.2
Zarqa	44(14.62)	14.00	+0.62
Balqa	18(5.98)	6.00	0.02
Madaba	6(1.99)	2.00	0.01
**South geographical region**			
Al-Kerak	8(2.66)	4.00	1.34
Aqaba	6(1.99)	2.00	0.01
Maan	6(1.99)	2.00	0.01
Tafilah	3(1)	1.00	0.00
**Gender**			
Male	154(51.16)	51.00	+0.16
Female	147(48.84)	49.00	0.16
**Age groups**			
18-24	54(17.94)	21.00	3.06
25-29	41(13.62)	11.00	+2.62
30-34	30(9.96)	10.00	0.04
35-39	28(9.30)	10.00	0.7
40-44	31(10.29)	10.00	+0.29
45-49	27(8.97)	9.00	0.03
50-54	28(9.30)	8.00	+1.30
55-59	18(5.98)	6.00	0.02
60-64	20(6.64)	5.00	+1.64
65+	24(7.97)	10.00	2.03

^*^ Department of Statistics [27]

Regarding health insurance, 77% of respondents reported being insured, with 43% covered by the Ministry of Health (MOH) . Health indicators showed that 28% of participants had current comorbidities, with 41% reporting two or more comorbidities[25]. More than half of the participants (57%) engaged in regular exercise, such as walking or other activities, with approximately 90% exercising between one and three hours or more weekly. Smoking was reported by 40% of participants, with cigarettes being the most common type. Additionally, 92% of participants were vaccinated against COVID-19, and 40% reported a previous COVID-19 infection (Table 2).

**Fig. 1:**
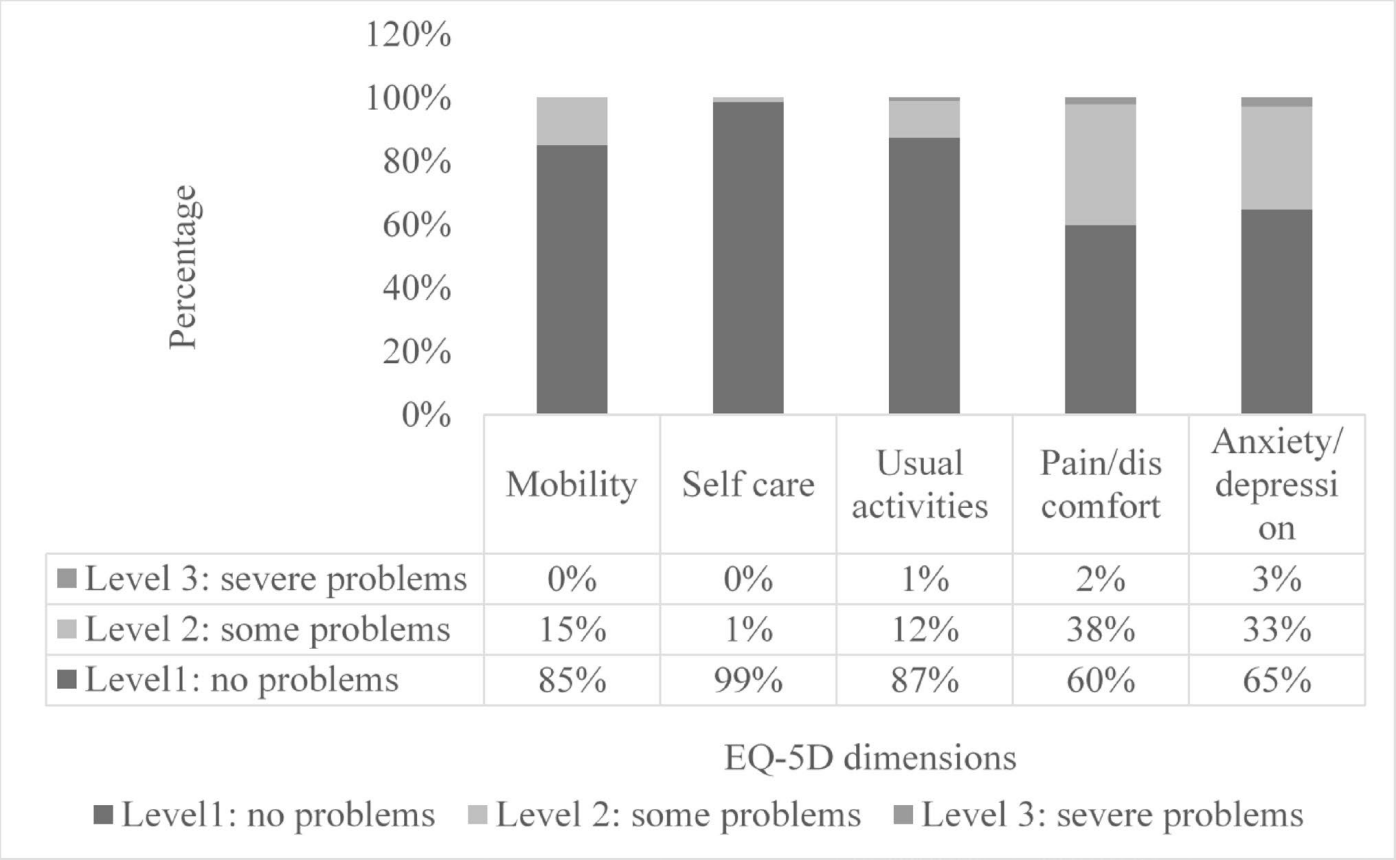
Distribution of EQ-5D-3L severity levels among respondents[25]

The mean EQ VAS score in the study population was 81 (SD ± 14)[25]. Forty percent of respondents reported a health profile without problems (i.e., health state “11,111”). The most frequently reported health problems were pain/discomfort, followed by anxiety/depression, while the least reported health problems were in self-care[25] (Fig. 1).

### Model’s assumptions test results

The assumption testing showed no multicollinearity among independent variables; the residuals were not normally distributed, but the histogram was symmetrical, and heteroskedasticity was present. Therefore, we corrected for heteroskedasticity in the multilevel mixed effects and random intercept models in our analysis.

### Purposeful selection results

In the univariate variable screening, factors with *p*-values ≤ 0.25 were included in the multivariable regression models. The multilevel mixed effects model identified four factors: marital status, employment status, having comorbidities, and COVID-19 vaccination. The Tobit random intercept model identified nine factors: gender, geographical region, education, marital status, employment status, health insurance status, smoking status, exercise status, and COVID-19 vaccination status. Additionally, the random intercept model identified nine factors: gender, geographical region, education, marital status, employment status, health insurance status, smoking status, exercise status, and COVID-19 vaccination status. Tables 3 summarize the detailed regression results, illustrating the relationship between each factor and cTTO.

**Table 3:** Comparison of adjusted univariate linear regression models for the associations of factors associated with cTTO scores (N = 301)

Characteristic	Multilevel mixed effects model ^a^Univariate Analysis			Random intercept model ^a^Univariate Analysis			Tobit random intercept model ^a^Univariate Analysis		
	β	SE	*P*-Value	β	SE	*P*-Value	β	SE	*P*-Value
Age, years (Ref:18–30, N = 105)									
31–45 (N = 84)	0.011	0.037	0.776	0.052	0.052	0.317	0.058	0.058	0.323
46–60 (N = 75)	-0.015	0.039	0.698	0.014	0.054	0.795	0.008	0.060	0.893
60 + (N = 37)	-0.055	0.049	0.258	0.029	0.068	0.673	0.012	0.076	0.872
Likelihood ratio test for joint significance			0.603			0.789			0.778
Gender (ref: Female, N = 147)									
Male (N = 154)	0.032	0.030	0.281	0.106	0.041	*0.009	0.117	0.045	*0.010
Marital status (ref: Single, N = 78)									
Married (N = 207)	− 0.020	0.034	0.562	− 0.031	0.047	0.516	− 0.039	0.053	0.453
Widowed /Divorced (N = 16)	− 0.175	0.070	0.012	− 0.171	0.098	0.075	− 0.183	0.109	0.092
Likelihood ratio test for joint significance			*0.042			*0.207			*0.240
Education (ref: Less than secondary, N = 21)									
Secondary (N = 79)	0.070	0.063	0.266	0.097	0.086	0.263	0.106	0.097	0.273
Intermediate diploma (N = 51)	0.078	0.067	0.240	0.130	0.091	0.154	0.137	0.102	0.179
Bachelor’s degree and above (N = 150)	0.062	0.060	0.299	0.191	0.082	0.020	0.204	0.092	0.026
Likelihood ratio test for joint significance			0.683			*0.054			*0.079
Employment status (ref: Employed, N = 154)									
Unemployed (N = 105)	− 0.103	0.032	0.001	− 0.161	0.044	0.00	− 0.179	0.049	0.000
Retired (N = 42)	− 0.018	0.044	0.683	0.098	0.060	0.103	0.099	0.067	0.141
Likelihood ratio test for joint significance			*0.006			*0.000			*0.000
Geographic region (ref: North region, N = 89)									
Middle region (N = 189)	− 0.048	0.033	0.144	− 0.130	0.045	0.004	− 0.140	0.050	0.006
South region (N = 23)	− 0.043	0.060	0.476	− 0.138	0.082	0.094	− 0.159	0.092	0.084
Likelihood ratio test for joint significance			0.350			*0.013			*0.017
Area of residence (ref: Rural, N = 22)									
Urban (N = 279)	0.021	0.057	0.707	0.036	0.079	0.649	0.054	0.088	0.541
Health insurance status (ref: No insurance, N = 70)									
Have insurance (N = 231)	0.016	0.035	0.638	0.080	0.048	*0.096	0.080	0.054	*0.138
Number of comorbidities (ref: None, N = 218)									
One comorbidly (N = 49)	− 0.005	0.040	0.897	− 0.028	0.056	0.618	− 0.032	-0.063	0.614
More than one comorbidity (N = 34)	− 0.111	0.047	0.018	− 0.048	0.066	0.466	− 0.080	0.073	0.275
Likelihood ratio test for joint significance			*0.060			0.711			0.521
Smoking status (ref: smoker, N = 121)									
Non-Smoker (N = 180)	0.010	0.030	0.729	0.054	0.042	*0.194	0.059	0.047	*0.210
Exercise status (ref: No, N = 130)									
Yes (N = 171)	0.028	0.030	0.340	0.080	0.041	*0.053	0.089	0.046	*0.055
EQ VAS (N = 300)	0.001	0.001	0.315	0.000	0.001	0.524	0.001	0.002	0.394
Reported COVID-19 infection status (Ref: No COVID-19, N = 180)									
Yes (N = 121)	− 0.006	0.030	0.836	0.037	0.042	0.383	0.040	0.047	0.389
Reported COVID-19 Vaccination status (Ref: No vaccination, N = 24)									
Yes (N = 277)	0.117	0.054	*0.031	0.141	0.075	*0.062	0.159	0.084	*0.060

^a^Adjusted for the severity of health states. β: regression coefficient; SE: standard error

^*^P < 0.25

The three models showed that married, widowed, and divorced respondents reported lower health state values than single respondents. Unemployed respondents valued health status lower than employed and retired respondents. Moreover, being vaccinated for COVID-19 was associated with higher health state values than being non-vaccinated (Table 3). On the other hand, only the multilevel mixed effects model showed that respondents with more than one comorbidity valued health states lower than those with one or no comorbidity. On the contrary, the random intercept and Tobit random intercept models showed that males, those with higher education, those with health insurance, those who exercise, and non-smokers value health states higher than females, those with lower education levels, those without health insurance, smokers, and those who do not exercise. Also, both models reported lower valuations of health states among respondents living in the middle and southern regions of Jordan compared with those living in the north region (Table 3).

The results of the purposeful selection showed that for the final multilevel mixed effects regression model, age categories, gender, geographical region, education level, and exercise status were identified as confounders and included in the multivariable regression analysis, along with employment, marital status, COVID-19 vaccination status, and comorbidities.

In the random intercept model, age and comorbidities were identified as confounders. Therefore, gender, age categories, education level, marital status, employment status, geographical region, health insurance, comorbidities, area type, smoking status, and exercising status were included in the final multivariable regression model.

For the final Tobit intercept model, after checking for confounders, we selected gender, age categories, education level, marital status, employment status, geographical region, health comorbidities, smoking status, and exercise status for inclusion in the multivariable regression model.

### Goodness of fit of models and model selection

The results showed that the multivariable multilevel mixed effects regression model provided the best fit for the data with the lowest AIC and BIC statistics, followed by the random intercept model (Table 4). We selected the multilevel mixed effects model to identify factors associated with cTTO valuation in our study because we were able to correct for heteroskedasticity, and it resulted in the lowest AIC and BIC results.

**Table 4:** Comparison of adjusted multivariable linear regression models for the associations of factors associated with cTTO scores (N = 301)

Characteristic	Multilevel mixed effects model ^a^Multivariable Analysis			Random intercept model ^a^Multivariable Analysis			Tobit random intercept model ^a^Multivariable Analysis		
	β	SE	*P*-Value	β	SE	P-Value	β	SE	P-Value
Age, years (Ref:18–30, N = 105)									
31–45 (N = 84)	0.009	0.042	0.834	0.093	0.060	0.116	0.102	0.070	0.146
46–60 (N = 75)	0.021	0.050	0.670	0.056	0.070	0.430	0.054	0.090	0.531
60 + (N = 37)	− 0.003	0.084	0.969	− 0.041	0.102	0.683	− 0.065	0.113	0.564
Gender (ref: Female, N = 147)									
Male (N = 154)	− 0.035	0.030	0.252	0.050	0.047	0.281	0.060	0.055	0.284
Marital status (ref: Single, N = 78)									
Married (N = 207)	− 0.020	0.040	0.619	− 0.100	0.056	0.071	− 0.111	0.068	0.104
Widowed /Divorced (N = 16)	− 0.154	0.107	0.151	− 0.137	0.120	0.258	− 0.133	0.124	0.282
Education (ref: Less than secondary, N = 21)									
Secondary (N = 79)	0.052	0.067	0.450	0.090	0.080	0.283	0.092	0.094	0.329
Intermediate diploma (N = 51)	0.031	0.079	0.697	0.100	0.093	0.298	0.098	0.099	0.322
Bachelor’s degree and above (N = 150)	− 0.004	0.0715	0.956	0.140	0.083	0.091	0.143	0.091	0.115
Employment status (ref: Employed, N = 154)									
Unemployed (N = 105)	− 0.110	0.038	*0.003	− 0.113	0.051	*0.025	− 0.130	0.060	*0.025
Retired (N = 42)	− 0.006	0.070	0.940	0.164	0.090	0.066	0.190	0.085	*0.0268
Geographic region (ref: North region, N = 89)									
Middle region (N = 189)	− 0.040	0.034	0.233	− 0.063	0.050	0.170	− 0.066	0.052	0.202
South region (N = 23)	− 0.058	0.062	0.350	− 0.147	− 0.087	0.093	− 0.172	0.090	0.054
Area of residence (ref: Rural, N = 22)									
Urban (N = 279)	^b^NPC	^b^NPC	^b^NPC	^b^NPC	^b^NPC	^b^NPC	^b^NPC	^b^NPC	^b^NPC
Health insurance status (ref: No insurance, N = 70)									
Have insurance (N = 231)	^b^NPC	^b^NPC	^b^NPC	^b^NPC	^b^NPC	^b^NPC	^b^NPC	^b^NPC	^b^NPC
Number of comorbidities (ref: None, N = 218)									
One comorbidly (N = 49)	0.022	0.039	0.581	0.019	0.056	0.740	0.021	0.070	0.757
More than one comorbidity (N = 34)	− 0.120	0.060	*0.047	− 0.083	0.080	0.296	− 0.114	0.084	0.176
Smoking status (ref: smoker, N = 121)									
Non-Smoker (N = 180)	^b^NPC	^b^NPC	^b^NPC	0.100	0.050	*0.029	0.114	0.049	*0.019
Exercise status (ref: No, N = 130)									
Yes (N = 171)	0.031	0.032	0.332	0.048	0.042	0.251	0.060	0.045	0.218
EQ VAS (N = 300)	^b^NPC	^b^NPC	^b^NPC	^b^NPC	^b^NPC	^b^NPC	^b^NPC	^b^NPC	^b^NPC
Reported COVID-19 infection status (Ref: No COVID-19, N = 180)									
Yes (N = 121)	^b^NPC	^b^NPC	^b^NPC	^b^NPC	^b^NPC	^b^NPC	^b^NPC	^b^NPC	^b^NPC
Reported COVID-19 Vaccination status (Ref: No vaccination, N = 24)									
Yes (N = 277)	0.080	0.061	0.185	^b^NPC	^b^NPC	^b^NPC	^b^NPC	^b^NPC	^b^NPC
Goodness of fit									
AIC	3358.458			3693.177			4541.113		
BIC	3496.681			3819.381			4667.316		

^a^Adjusted for severity of health states. ^b^NPC: Not potential candidate (variable was not identified during purposeful selection as a potential predictor). β: Regression coefficient; SE: Standard error; AIC: Akaike Information Criterion; BIC: Bayesian Information Criterion. *P < 0.05

### Results of the final multivariable regression analysis

After adjusting for health state severity, the multivariable mixed model showed a statistically significant negative relationship (*p* < 0.05) between employment status, comorbidities, and health state valuation. Unemployed individuals and participants having more than one comorbidity value health states lower than those of employed individuals and those without comorbidities, provided all other factors remained constant (Table 4).

On average, females, those aged 31–45 and 46–60, respondents who exercise, COVID-19 vaccinated, and those with an intermediate diploma showed higher valuations than males, respondents in age groups 18–30, and above 45 years old, those who do not exercise, respondents with lower education levels, and those who were not vaccinated for COVID-19. On the other hand, married and widowed/divorced respondents aged 60 and above, and those living in the middle and south regions of Jordan, showed lower health state valuations than single respondents, those aged 18–30, and those living in the north region of Jordan. However, none of these variables were significant factors of cTTO (Table 4).

## Discussion

This study aimed to identify factors that are associated with health state valuation among the Jordanian population elicited using the cTTO method. The results showed that employment status and having more than one comorbidity were significantly associated with health state valuation in Jordan.

Unemployment was associated with lower stated health state values, and this relationship was statistically significant in all models tested. The negative consequences of unemployment for individuals and society can explain this finding. Evidence suggests that unemployment negatively affects health-related quality of life[41]. It reduces QALYs by 0.096 compared with employed individuals [41]. Also, unemployment interferes with usual activities and may make people less active [42]. For example, it is associated with depression and mental health problems [43, 44].

Furthermore, unemployment is closely linked to poverty and affects nations’ productivity, particularly when the unemployment rate is high among the young. A closer examination of the Jordanian economy reveals that unemployment and poverty are the primary economic challenges [45]. During our valuation study implementation period, the reported unemployment rate for 2021 was 23.3% [27]. During the period 2020–2022, the COVID-19 pandemic’s economic impact on Jordan was evident, contributing to a rise in the unemployment rate [46]. Compared to the European Union (EU), Jordan’s unemployment rate is approximately five times higher than the EU average [47]. The economic challenge of high unemployment rates in Jordan provides the context within which our study respondents valued health states.

We have looked deeply into the unemployment status stratified by gender and health insurance. Not having health insurance can still contribute to low health state values among the unemployed. We found that 36% of the unemployed respondents did not have health insurance, making them more prone to trade off more life years to avoid health problems they might be unable to treat [48, 49]. Despite the development of the Jordanian healthcare system, health disparities in access to healthcare services remain a significant challenge [50]. Jordan is committed to achieving universal health coverage (UHC) by 2030 [51, 52], which will protect underprivileged people when they face health problems by ensuring they receive the necessary treatment regardless of their financial situation [52].

Moreover, we found that 80% of unemployed respondents were females. This finding reflects the Jordanian labor market, where only 15% of women were reported to be economically productive in 2018 [53, 54]. Females in Jordan, like those in other Arab countries, are often responsible for providing informal caregiving to ill family members, in addition to parenting and other roles [55]. Their multiple roles make females trade off more life years to avoid critical health situations for family members[17]. Gender was found to be a confounder of the association between employment and the valuation of health states. Therefore, it was included in the final multivariable model.

As unemployment is associated with lower health state values, our results may also indicate the need to carefully assess equity implications, including the efficiency-equity tradeoff in HTA, including the use of distributional cost-effectiveness analysis. CUA does not provide an estimate of the impact of new health technologies, like new medicines, on health inequalities [56, 57]. The implication of an equal distribution of the costs and benefits of new technologies across society means that both employed and non-employed individuals can benefit equally from these new health interventions. However, this might not be the case because the unemployed may not have insurance, and, according to our study results, the unemployed do not prefer health technologies that improve the length of life over the quality of life. For example, if we have an unemployed caregiver in Jordan, they need more interventions that improve their HRQoL. Failing to capture the spillover effects of health technologies might exacerbate inequality among the unemployed population.

In contrast to previous studies, having more than one comorbidity was significantly associated with health state cTTO values. Respondents with more than one comorbidity valued health states lower than those without comorbidities. However, this result should be interpreted with caution as the sample size of respondents with more than one comorbidity was small, and the resulting p-value from the multilevel mixed effects regression model was close to 0.05 [58, 59].

Some previously published studies have identified age, gender, geographical region, education, and marital status as determinants of health state valuations in Egypt, the UK, Canada, Brazil, and Poland [17, 18, 20, 60–62]. In our study, those factors did not show a strong statistical association with health state valuation. However, they were identified as potential confounders, and we therefore kept them in the final multivariable model because even removing confounders with weak statistical correlations could have introduced bias. For instance, education is a confounder for the relationship between employment and valuation of health states. The inclusion of education reduced the coefficient for unemployment in the multivariable regression model from -0.083 to -0.099. According to the change-in-estimate philosophy [33], this means that education is associated with both employment (a factor) and the valuation of health states (an outcome). Those who are unemployed are less educated than the employed, and the true effect of education on employment is captured by including it in the multivariable model. Moreover, our findings highlight the significance of the study context and the varying values placed on health states across different countries.

### Recommendations and policy implications

One policy implication of our study’s results is the choice of perspective in economic evaluations. For instance, a provider or payer perspective in economic evaluations will not take into consideration the loss of productivity due to unemployment, and as our results showed that unemployment is associated with lower cTTO values, which means that the unemployed in Jordan are willing to trade off more life years, which might be their productive years. Therefore, it might be useful to assess the feasibility of adopting a societal perspective in economic evaluation studies in Jordan. However, this requires investment in data collection and capacity building to calculate indirect costs.

The statistically non-significant associations between most of the sociodemographic characteristics, some health-related factors, and cTTO health states’ valuations in our study suggest that the impact of respondents’ characteristics on cTTO valuations is small. This finding is consistent with the results reported by van Nooten et al. (2018) and Santos et al. (2020) [19, 20]. During the cTTO experiments, respondents will interact with scenarios that trigger emotions and thoughts influenced by their value systems. Further investigations are needed to explain the variation of cTTO valuations by examining respondents’ personality traits and how they interact with cTTO valuation tasks within their national cultures. Furthermore, conducting additional qualitative studies to understand how people value health states may be needed along quantitative research [19, 20, 63].

### Strengths and limitations

Our study encountered several strengths. First, we used the cTTO data from the Jordanian EQ-5D-3L valuation study[25]. Second, our study was representative of the Jordanian population, and high-quality assurance standards were applied in accordance with the standardized EuroQol EQ-VT v2.1 valuation protocol [15, 16]. Furthermore, we collected not only socioeconomic characteristics but also information on health-related indicators, such as smoking and exercise.

On the other hand, we have encountered several limitations: representativeness is tied to what is known and measurable. Our quota sampling did not include employment, education, and marital status as stratification factors in addition to geographical region, age, and gender. In addition, we agree with van Nooten et al. (2018) that there is a need to systematically investigate the impact of respondents’ socio-demographics on cTTO health state valuation by designing a focused study with larger sample sizes rather than using by-product data from valuation studies [19]. Despite this limitation, future valuation studies in Jordan can benefit from this study in sampling planning.

Another limitation may be the respondent’s understanding of exercise. We kept it a general question, without differentiating between going to the gym or engaging in any physical activity, such as walking. We did not ask about the number of family members in a household or the number of children per family, even though previous studies have shown these variables to be predictors of health state valuations [19, 64].

## Conclusion

Employment status is associated with health states valuation in Jordan. Unemployed persons appear to place relatively greater value on good health than on additional life years compared with employed persons. Furthermore, sociodemographic characteristics may have a relatively small impact on health state valuation in Jordan. Future research should explore cultural dimensions and personality traits to better understand how individuals value health states.

## Electronic supplementary material

Below is the link to the electronic supplementary material.

Supplementary file 1 (DOCX 19 kb)

## Data Availability

Data from the corresponding author upon a reasonable request.
