# Supplementary material for: Factors associated with health state valuations: a secondary analysis of an EQ-5D-3L valuation study from Jordan
Source: Qual Life Res. 2026 May 3;35(6):154. doi: 10.1007/s11136-025-04151-2 (PMC13136213; doi:10.1007/s11136-025-04151-2)
Supplement: Supplementary file 1 — (DOCX 19 kb) [file 11136_2025_4151_MOESM1_ESM.docx]

**Supplementary material**

**Table 1. Independent Variables (Factors)**

| **Factors** | **Description** | **Categories** |
| --- | --- | --- |
| Demographic Variables | Age  Gender | 31-45 |
|  |  | 46-60 |
| Socioeconomic Variables | Marital status | 60+ |
|  | Education level | Less than secondary  Secondary  Intermediate diploma  Bachelor’s degree and above |
|  | Employment status | Employed  Unemployed  Retired |
| Geographic and Area Variables | Geographic area | North of Jordan (Irbid, Al-Mafraq, Jerash, Ajloun)  Middle of Jordan (Amman, Zarqa, Balqa, Madaba)  South of Jordan (Al-Kerak, Aqaba, Ma’an, Tafilah) |
| Health-Related Variables | Health insurance | Have health insurance  No health insurance |
|  | Number of *comorbidities | No comorbidities  One comorbidity  Two comorbidities |
|  | Smoking status | Smoker  Non-smoker |
|  | Exercise status | Exercise  No exercise |
|  | COVID-19 infection status | Infected  Not infected |
|  | COVID-19 vaccination status | Vaccinated  Not vaccinated |

Comorbidities: defined as self-reporting of one chronic diseases or more
